# The Association Between Clinical Characteristics and Motor Symptom Laterality in Patients With Parkinson's Disease

**DOI:** 10.3389/fneur.2021.663232

**Published:** 2021-05-31

**Authors:** Sha Zhu, Min Zhong, Yu Bai, Zhuang Wu, Ruxin Gu, Xu Jiang, Bo Shen, Jun Zhu, Yang Pan, Jingde Dong, Pingyi Xu, Jun Yan, Li Zhang

**Affiliations:** ^1^Department of Geriatrics, Nanjing Brain Hospital Affiliated to Nanjing Medical University, Nanjing, China; ^2^Department of Biological Sciences, University of Toronto Scarborough, Toronto, ON, Canada; ^3^Department of Neurology, First Affiliated Hospital of Guangzhou Medical University, Guangzhou, China

**Keywords:** Parkinson's disease, asymmetry, motor, non-motor symptoms, wearable sensors, gait analysis

## Abstract

**Background and Purpose:** The unilateral onset and persistent asymmetry of motor symptoms are important characteristics of Parkinson's disease (PD). By using scales and wearable sensors, this study explored whether motor symptom laterality could affect non-motor symptom and gait performance.

**Methods:** A total of 130 right-handed patients with PD were enrolled in our study and were divided into two groups according to the side of predominant motor symptom presentation by using the Unified Parkinson's Disease Rating Scale part III. We measured the non-motor symptoms with the Non-motor symptoms Scale, sleep quality with the Parkinson's Disease Sleep Scale and Pittsburgh sleep quality index, cognitive function with the Mini-mental State Examination and Montreal Cognitive Assessment, quality of life with the Parkinson's Disease Questionnaire-39, and the severity of anxiety and depression with the Hamilton Anxiety Scale and Hamilton Depression Scale, respectively. All participants underwent the instrumented stand and walk test, and gait data were collected using a set of JiBuEn gait analysis system.

**Results:** We observed that left-dominant symptom PD patients (LPD) were associated with a greater impairment of sleep quality than right-dominant symptom PD patients (RPD). We found no difference between LPD and RPD in terms of gait performance. However, compared with the severe asymmetry RPD patients (RPD-S), severe asymmetry LPD patients (LPD-S) showed a shorter stride length and decreased range of motion of hip joints.

**Conclusions:** In this study, LPD was associated with a more severe sleep-related dysfunction than RPD. In addition, LPD-S exhibited more gait impairments than RPD-S. Considering that motor symptom laterality may affect the non-motor symptom and gait performance, it should be taken into account when evaluating and treating PD patients.

## Introduction

Parkinson's disease (PD) is a common neurodegenerative disease, which is mainly characterized by movement disorders, including bradykinesia, resting tremor, rigidity, and postural instability (1). The unilateral onset of PD and the persistent asymmetry of these main motor symptoms are important features that distinguish PD from other parkinsonian syndromes (2). Only ~16.4% of PD patients have shown bilateral symmetrical motor symptoms, while the proportion of multiple system atrophy-Parkinsonism is 48.3%, and the proportion of PSP is 52.9% (3). Previous studies have shown that compared with right-dominant symptom PD patients (RPD), left-dominant symptom PD patients (LPD) scored worse in the Non-motor symptoms Scale (NMSS) assessment (4). Left-onset PD patients performed worse in feedback-based associative learning than right-onset PD patients, and this condition may be related to the abnormal function of the right dorsal rostral putamen (5). An arterial spin labeling study showed that LPD experienced more severe perfusion changes in disease severity and visual-spatial dysfunction related areas (6). Moreover, white matter integrity has remarkably changed in RPD, but not in LPD, indicating that LPD may be related to a better prognosis than RPD (7). Hence, motor symptom laterality plays an important role in the clinical characteristics of patients with PD. However, limited information is available about the effect of motor symptom laterality on patients' gait performance. Although the UPDRS III score is a widely used clinical examination to evaluate motor function in PD, it only partially reflects the motor function in daily life and suffers from intra- and inter-rater reliability (8, 9). It is very limited for quantitative information on gait impairment. With the development of micro-electronics technology, the use of wearable sensors allows the quantitative analysis of the gait parameter of patients with PD (10, 11), which makes up for this deficiency and can find small changes that UPDRS III cannot reflect. Accordingly, this study aimed to explore the effect of motor symptom laterality on gait characteristics of PD patients by using wearable devices and assessing patients' non-motor symptoms. We speculate that the burden of gait characteristics and non-motor symptoms would be higher in LPD than RPD. In-depth knowledge of both non-motor symptoms and specific gait characteristics differences between LPD and RPD will aid the management of patients with PD.

## Methods

### Participants

**Figure 1:**
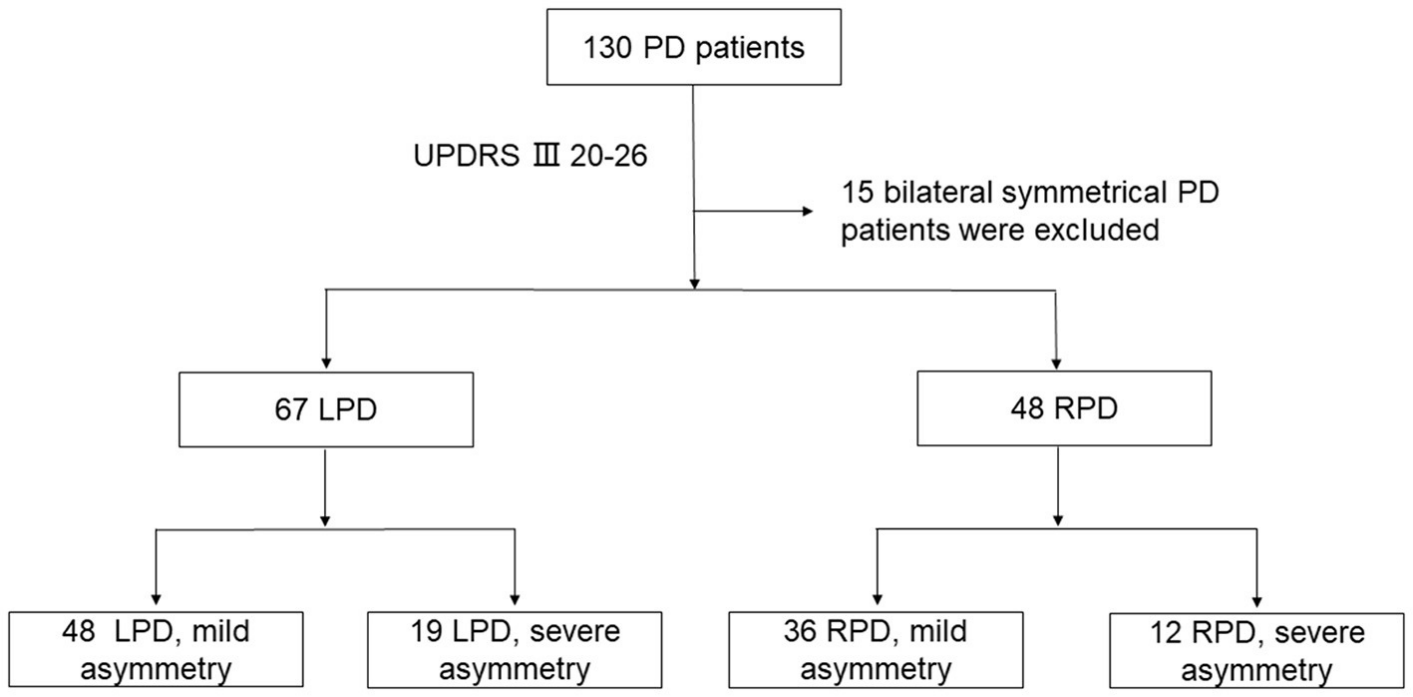
Study flow chart. PD, Parkinson's disease; LPD, left-dominant symptom PD patients; RPD, right-dominant symptom PD patients; UPDRS III, Unified Parkinson's Disease Rating Scale part 3.

In our research, we enrolled 130 patients from the Department of Geriatrics, Nanjing Brain Hospital Affiliated to Nanjing Medical University from October 2018 to November 2020. All patients were diagnosed with PD according to the Movement Disorder Society criteria (12). We excluded patients with cerebrovascular, spinal column, and musculoskeletal disease, which may influence gait performance. All participants were required to be right-handed (the dominant hand was defined as the hand used for writing). The Medical Ethics Committee of the Nanjing Brain Hospital Affiliated to Nanjing Medical University approved this study. All patients signed a written informed consent before the study. Unified Parkinson's Disease Rating Scale part III (UPDRS-III) (13) was employed to evaluate PD motor symptoms. We calculated clinical laterality by measuring the difference between the right and left UPDRS-III sub scores, by using questions 20–26. Subsequently, the idiopathic PD patients were grouped into LPD and RPD. To measure the degree of clinical laterality, we employed the following two groups (14): (1) mild right asymmetry defined as the total score of right bradykinesia, tremor, and rigidity divided by the left side total score >1 and <3, the same goes for the left side and (2) severe right or left asymmetry (named RPD-S and LPD-S, respectively), defined as the total score of bradykinesia, tremor, and rigidity on the right or left divided by the total score of the left or right ≥3. Fifteen participants with an asymmetry quotient = 1 were excluded because of their symmetric impairment of motor symptoms (Figure 1).

### Clinical Evaluation

The following baseline data of all the participants were collected: gender, age, height, weight, and the duration of the disease. Hoehn & Yahr (H-Y) stage and UPDRS were used to assess the motor symptoms. To assess the non-motor symptoms, we used the Non-motor symptoms Scale (NMSS). Parkinson's Disease Sleep Scale (PDSS) and Pittsburgh sleep quality index (PSQI) were applied to evaluate the patients' sleep quality. Cognitive function was assessed using the Montreal Cognitive Assessment (MoCA) and Mini-mental State Examination (MMSE). The quality of life was evaluated using the Parkinson's Disease Questionnaire-39 (PDQ39). Severity of anxiety and depression were quantified by the Hamilton Depression Scale (HAMD) and Hamilton Anxiety Scale (HAMA), respectively.

### Gait Data Collection

In our study, we used a set of JiBuEn gait analysis system to collect gait data (15). The accuracy of this equipment has been verified. All participants have completed the instrumented stand and walk test (16): Firstly, all of the participants were instructed to stand for 30 s, walk for 7 meters in a comfortable way, turn 180°, and finally walk back to the initial place. In this process, we used the JiBuEn gait analysis system to collect gait parameters.

### Statistical Analysis

We analyzed the data by using the statistical package SPSS v. 25.0. Data were expressed as mean ± standard deviation (SD), and a *P* < 0.05 indicated significant difference. Kolmogorov–Smirnov test was initially used to check the normal distribution of quantitative data. Two-sample *t*-tests were used when both sets of data followed a normal distribution; otherwise, the Mann–Whitney *U*-Test was used. Chi-square test was used for qualitative data. The following formula (1) was used to compute for the variability of gait parameters, and then, integrated using formula (2) (17, 18). We also used the asymmetry index (AI) to evaluate the symmetry of gait parameters by using formula (3) (19, 20).

(1) $$\begin{array}{l} {\text{CV}}_{\text{separate}}=\text{SD}÷\text{mean value} \end{array}$$

(2) $$\begin{array}{l} {\text{CV}}_{\text{combined}=}\sqrt{\frac{\text{C}{\text{V}}_{L}+{\text{CV}}_{R}}{2}}*\;100 \end{array}$$

CV indicates the coefficient of variation, and the subscripts L and R mean the left and right sides of patients, respectively.

(3) $$\begin{array}{l} \text{AI}=\frac{\text{max}\left({\text{X}}_{L},\;{\text{X}}_{R}\right)-\text{min}\left({\text{X}}_{L},\;{\text{X}}_{R}\right)}{\text{max }\left({\text{X}}_{L}\;,\;{\text{X}}_{R}\right)}*100 \end{array}$$

where X = [SL, ST, SwPT, StPT, HS, TO, ROM-AJ, ROM-KJ, ROM-HJ], and the subscripts L and R indicate the left and right sides of patients, respectively. SL, stride length; ST, stride time; SwPT, swing phase time; StPT, stance phase time; HS, heel strike angle; TO, toe-off angle; ROM, range of motion; AJ, ankle joint; KJ, knee joint; HJ, hip joint.

## Results

### Clinical Baseline Data of Participants

In our research, a total of 115 patients with PD were grouped into two groups, and their baseline data are listed in Table 1. No statistical difference was observed from all baseline data between the two groups.

**Table 1:** Clinical baseline data of patients.

	**LPD**	**RPD**	***P***	**LPD-S**	**RPD-S**	***P***
N	67	48		19	12	
Age (years)	65.72 ± 9.70	67.19 ± 8.56	0.402	64.68 ± 11.81	62.75 ± 9.54	0.823
Height (cm)	163.70 ± 7.00	165.75 ± 7.64	0.139	163.47 ± 7.59	167.42 ± 5.66	0.133
Weight (kg)	65.26 ± 10.37	65.94 ± 10.69	0.644	63.11 ± 9.88	70.13 ± 9.01	0.056
Male (%)	36 (53.73)	28 (58.33)	0.624	7 (36.84)	5 (41.67)	0.789
Education (%)			0.681			0.237
Illiteracy	15 (22.39)	8(16.67)		3 (15.79)	0(0.00)	
Primary school	9 (13.43)	10 (20.83)		2 (10.52)	1 (8.33)	
Middle school	33 (49.25)	24 (50.00)		9 (47.37)	9 (75.00)	
College	10 (14.93)	6 (12.5)		5 (26.32)	2 (16.67)	
PD duration (years)	5.79 ± 7.25	5.83 ± 4.27	0.235	2.71 ± 2.33	4.71 ± 3.73	0.116
Hoehn-Yahr stage	2.43 ± 0.86	2.43 ± 0.84	0.974	1.63 ± 0.52	1.92 ± 0.51	0.132
UPDRS III total scores	32.52 ± 16.07	33.46 ± 15.98	0.758	18.32 ± 6.84	23.25 ± 7.44	0.068

*Data were shown as mean ± SD*.

*PD, Parkinson's disease; LPD, left-dominant symptom PD patients; RPD, right-dominant symptom PD patients; LPD-S, severe asymmetry LPD; RPD-S, severe asymmetry RPD; UPDRS III, Unified Parkinson's Disease Rating Scale part 3*.

### Non-motor Symptoms

In this research, non-motor symptoms including NMSS, PDQ-39, MoCA, MMSE, HAMD, and HAMA were similar in both groups. However, compared with the LPD group, the RPD group scored better in PDSS (101.82 ± 25.48 vs. 118.60 ± 19.93, *P* < 0.001) and PSQI (7.48 ± 5.02 vs. 4.79 ± 4.01, *P* = 0.003), and statistical difference was observed between LPD-S and RPD-S for PDSS (108.26 ± 24.38 vs. 123.75 ± 15.40, *P* = 0.039). All related data were listed in Table 2.

**Table 2:** Non-motor symptoms of participants.

	**LPD**	**RPD**	***P***	**LPD-S**	**RPD-S**	***P***
UPDRS I	2.65 ± 2.00	2.79 ± 1.89	0.745	3.32 ± 2.43	2.33 ± 1.37	0.307
UPDRS II	13.58 ± 6.01	15.49 ± 7.24	0.326	9.53 ± 4.41	12.00 ± 4.67	0.148
MMSE	25.55 ± 4.14	24.70 ± 5.79	0.815	27.00 ± 3.09	27.17 ± 5.67	0.247
MoCA	22.20 ± 5.73	21.13 ± 7.32	0.686	24.42 ± 5.26	25.75 ± 5.88	0.402
HAMD	8.24 ± 7.16	8.04 ± 5.91	0.977	8.47 ± 9.08	8.00 ± 4.13	0.542
HAMA	7.76 ± 6.24	7.19 ± 5.48	0.716	7.42 ± 6.74	8.42 ± 4.38	0.653
NMSS	11.23 ± 5.22	11.28 ± 5.01	0.921	9.47 ± 5.69	11.08 ± 4.68	0.419
PDSS	101.82 ± 25.48	118.60 ± 19.93	** <0.001**	108.26 ± 24.38	123.75 ± 15.40	**0.039**
PSQI	7.48 ± 5.02	4.79 ± 4.01	**0.003**	7.63 ± 5.73	4.50 ± 3.66	0.121
PDQ39	34.82 ± 27.67	38.72 ± 29.45	0.486	24.16 ± 20.99	28.08 ± 18.45	0.516

*Data were shown as mean ± SD; Bold font indicated significant results*.

*PD, Parkinson's disease; LPD, left-dominant symptom PD patients; RPD, right-dominant symptom PD patients; LPD-S, severe asymmetry LPD; RPD-S, severe asymmetry RPD; UPDRS I, Unified Parkinson's Disease Rating Scale part 1; UPDRS II, Unified Parkinson's Disease Rating Scale part 2; MMSE, Mini-mental State Examination; MoCA, Montreal Cognitive Assessment; HAMD, Hamilton Depression Scale; HAMA, Hamilton Anxiety Scale; NMSS, Non-motor symptoms Scale; PDSS, Parkinson's Disease Sleep Scale; PSQI, Pittsburgh sleep quality index; PDQ39, Parkinson's Disease Questionnaire-39*.

### Spatiotemporal Gait Parameters

The following spatiotemporal gait parameters were collected in our research (Table 3): stride length (SL), gait velocity (GV), cadence (CA), stride time (ST), stance phase time (StPT), and swing phase time (SwPT). We also measured SL variability (CV-SL), ST variability (CV-ST), StPT variability (CV-StPT), and SwPT variability (CV-SwPT). We observed no difference between LPD and RPD. However, compared with RPD-S group, the LPD-S group exhibited a shorter SL (0.99 ± 0.19 vs. 1.12 ± 0.11, *P* = 0.036).

**Table 3:** Spatiotemporal gait parameters of participants.

	**LPD**	**RPD**	***P***	**LPD-S**	**RPD-S**	***P***
SL (m)	0.92 ± 0.25	0.94 ± 0.25	0.580	0.99 ± 0.19	1.12 ± 0.11	**0.036**
GV (m/s)	0.73 ± 0.25	0.75 ± 0.23	0.829	0.80 ± 0.22	0.86 ± 0.18	0.408
CA (steps/min)	94.66 ± 19.98	95.61 ± 18.85	0.437	96.18 ± 18.47	92.23 ± 17.44	0.559
ST (s)	1.42 ± 0.98	1.30 ± 0.25	0.432	1.30 ± 0.32	1.34 ± 0.22	0.187
StPT (%)	65.64 ± 5.97	65.17 ± 6.44	0.632	64.84 ± 6.00	65.63 ± 4.17	0.694
SwPT (%)	34.38 ± 5.97	34.54 ± 6.22	0.446	35.17 ± 6.01	34.39 ± 4.19	0.695
StPT (s)	0.98 ± 0.93	0.86 ± 0.23	0.475	0.86 ± 0.31	0.89 ± 0.20	0.273
SwPT (s)	0.44 ± 0.07	0.44 ± 0.04	0.529	0.44 ± 0.03	0.45 ± 0.03	0.391
CV-SL	27.47 ± 8.06	28.37 ± 9.18	0.372	25.15 ± 5.76	23.11 ± 4.49	0.307
CV-ST	24.30 ± 10.68	26.24 ± 12.31	0.598	23.46 ± 7.52	23.44 ± 5.41	0.598
CV-StPT	17.50 ± 5.18	18.27 ± 6.75	0.839	17.30 ± 5.72	16.44 ± 3.35	0.839
CV-SwPT	24.18 ± 9.00	25.48 ± 11.37	0.746	23.26 ± 8.75	22.00 ± 4.42	0.746

*Data were listed as mean ± SD; Bold font indicated significant results*.

*PD, Parkinson's disease; LPD, left-dominant symptom PD patients; RPD, right-dominant symptom PD patients; LPD-S, severe asymmetry LPD; RPD-S, severe asymmetry RPD; SL, stride length; CA, cadence; GV, gait velocity; ST, stride time; StPT, stance phase time; SwPT, swing phase time; CV, coefficient of variation*.

### Kinematic Gait Parameters

Heel strike (HS) and toe-off (TO) angles were measured in our study. In addition, we evaluated the range of motion (ROM) of ankle joints (ROM-AJ), knee joints (ROM-KJ), and hip joints (ROM-HJ) in our research (Table 4). We measured ROM by calculating the different value between the maximum and minimum angles of the joints mentioned above in the sagittal plane. The ROM values were taken from the averages of the left and right sides to reflect the overall range of motion of the three joints. We did not observe a remarkable difference in HS, TO, ROM-AJ, ROM-HJ, and ROM-KJ between the LPD and RPD. However, compared with RPD-S group, the LPD-S group exhibited a decreased ROM-HJ (34.15 ± 6.40 vs. 38.88 ± 5.10, *P* = 0.039).

**Table 4:** Kinematic gait parameters of participants.

	**LPD**	**RPD**	***P***	**LPD-S**	**RPD-S**	***P***
HS (°)	12.07 ± 4.29	11.81 ± 3.81	0.745	13.64 ± 4.21	14.43 ± 2.67	0.563
TO (°)	5.24 ± 1.21	5.36 ± 1.30	0.618	5.56 ± 1.02	5.43 ± 0.85	0.724
ROM-AJ (°)	21.60 ± 6.00	23.34 ± 5.54	0.116	23.91 ± 5.80	25.18 ± 3.78	0.508
ROM-KJ (°)	51.07 ± 13.64	50.21 ± 13.33	0.738	57.04 ± 12.55	57.65 ± 10.20	0.888
ROM-HJ (°)	32.02 ± 8.68	32.33 ± 8.94	0.853	34.15 ± 6.40	38.88 ± 5.10	**0.039**

*Data were listed as mean ± SD; Bold font indicated significant results*.

*PD, Parkinson's disease; LPD, left-dominant symptom PD patients; RPD, right-dominant symptom PD patients; LPD-S, severe asymmetry LPD; RPD-S, severe asymmetry RPD; HS, heel strike angle; TO, toe-off angle; ROM, range of motion; AJ, ankle joint; KJ, knee joint; HJ, hip joint*.

### Symmetry Analysis of Gait Parameters

Gait symmetry data were provided in Table 5, and no significant difference was found for all symmetry related indicators.

**Table 5:** Symmetry analysis of gait parameters.

	**LPD**	**RPD**	***P***	**LPD-S**	**RPD-S**	***P***
AI-SL	3.34 ± 2.97	2.77 ± 2.09	0.077	2.30 ± 0.91	2.55 ± 0.67	0.430
AI-ST	10.78 ± 14.20	11.54 ± 11.73	0.426	11.33 ± 9.97	9.02 ± 9.73	0.429
AI-StPT (%)	6.23 ± 7.59	7.91 ± 12.79	0.550	5.59 ± 6.09	5.60 ± 3.90	0.491
AI-SwPT (%)	11.20 ± 12.29	11.08 ± 10.68	0.934	9.71 ± 9.00	10.23 ± 7.16	0.996
AI-StPT (s)	14.77 ± 16.57	16.59 ± 18.15	0.618	15.50 ± 13.11	12.82 ± 11.79	0.598
AI-SwPT (s)	9.31 ± 11.24	8.70 ± 6.68	0.496	7.34 ± 6.07	8.33 ± 5.66	0.417
AI-HS	17.61 ± 12.31	18.97 ± 13.58	0.574	15.13 ± 8.67	14.69 ± 10.98	0.900
AI-TO	10.31 ± 10.31	10.79 ± 8.93	0.485	9.20 ± 6.87	8.37 ± 5.77	0.732
AI-ROM-AJ	10.35 ± 8.43	11.88 ± 7.54	0.136	8.78 ± 8.07	10.65 ± 8.65	0.546
AI-ROM-KJ	17.05 ± 14.33	16.10 ± 13.28	0.729	17.22 ± 16.58	11.77 ± 10.70	0.311
AI-ROM-HJ	13.00 ± 14.16	15.11 ± 13.48	0.188	13.30 ± 12.42	12.19 ± 10.23	0.796

*Data were shown as mean ± SD*.

*PD, Parkinson's disease; LPD, left-dominant symptom PD patients; RPD, right-dominant symptom PD patients; LPD-S, severe asymmetry LPD; RPD-S, severe asymmetry RPD; AI, asymmetry index; SL, stride length; ST, stride time; StPT, stance phase time; SwPT, swing phase time; HS, heel strike angle; TO, toe-off angle; ROM, range of motion; AJ, ankle joint; KJ, knee joint; HJ, hip joint*.

## Discussion

In this study, we enrolled 130 patients to assess the patients' non-motor symptoms, and explored the effect of motor symptom laterality on the gait characteristics of PD patients by using wearable devices. In-depth knowledge of both the non-motor symptoms and specific gait characteristics differences between LPD and RPD will aid in the management of PD patients.

### Differences in Non-motor Symptoms Between LPD and RPD

In our study, we found that LPD showed a more severe sleep-related dysfunction than RPD. This result is similar to the previous study, which indicated that left-onset PD patients suffered more daytime dozing and nocturnal hallucinations (21). Recent study found no difference between LPD and RPD in PDSS, but they found that LPD scored worse especially in the domain of sleep/fatigue in the NMSS assessment (4). We tentatively atrributed this condition to the following points: firstly, the brain exhibited an uneven distribution pattern of dopamine in healthy individuals. It was reported that the level of dopamine in the striatum of the left hemisphere was higher than that of the right hemisphere (22). Further autopsy studies have supported the occurrence of dopamine asymmetry (23). As a result, compared with LPD, RPD have more dopaminergic neural reserves to deal with PD-related pathological changes. Secondly, the neural network in the right hemisphere is associated with the levels of vigilance and awakening in healthy people (24, 25). The vigilance function of the right hemisphere is especially sensitive to sleep deprivation (26), thus, changes in the right hemisphere neural networks in LPD may account for a more severe sleep-related dysfunction than RPD.

It was reported that LPD is asssociated with a higher overall non-motor impairment than RPD (4). This finding is inconsistent with our results. However, they did not consider the effect of the dominant hand as handedness is the most apparent asymmetry of human behavior (27), and a previous research has revealed that handedness is associated with motor symptom asymmetry (28). In addition, their disease duration was longer than ours. The frequency of non-motor symptom increases along with the duration of the disease (29) and some non-motor symptoms that may be related to asymmetry may not yet appear. We also found no difference between LPD and RPD in the cognitive function, which supports previous studies (30, 31). Considering that our sample size is relatively small, more research is needed. Further studies are also required for the subdomains of non-motor symptom and cognitive function.

### Differences in Gait Performance Between LPD and RPD

It has been well-known that patients with PD demonstrated a smaller step length (32, 33), slower gait velocity (34), and more variable gait pattern (20, 35, 36) than healthy people. However, little is known about the specific differences of gait performance between LPD and RPD. To address this gap, we designed this study. We made a comprehensive gait analysis of LPD and RPD from the perspectives of the spatiotemporal gait parameters and gait variability, kinematic gait parameters, and symmetry analysis of gait parameters. No difference was found between LPD and RPD for all gait parameters. However, of the 28 gait comparisons performed in the severe laterality group, two of these comparisons showed statistically significant differences. LPD-S group exhibited a shorter stride length and a smaller ROM-HJ than RPD-S. Stride length was regarded as the most prominent gait parameter in patients with PD (37). In addition, a previous study showed that a shorter stride length was associated with imbalance (38, 39). From this perspective, the LPD-S group may face a higher risk of falling than the RPD-S group. A smaller ROM-HJ indicates a more rigid gait pattern. Interestingly, this finding is consistent with a recent research which demonstrated that LPD presented with a mild but significantly higher motor impairment (4).

We expanded the scope of their research and our research located this mild but significant motor impairment in short stride length and decreased hip joint movement.

Our study also has some limitations. First of all, this research was not a *de novo* group, and some participants had taken anti-PD drugs before. However, by stopping antiparkinsonian medication for 24 h (72 h for controlled release anti-PD drugs), we minimized the effect of drug intervention. Secondly, in this study, the assessment of the dominant hand mainly depends on the patient's self-report, rather than a systematic scale, such as the Edinburgh Handedness Inventory (40). Finally, correction for multiple comparisons have also been argued because of the risk of incorrect dismissal of meaningful results, and considering our relatively small sample size, our findings have to be considered as preliminary results, more researches are needed to formalize this result.

In conclusion, despite the limitation we stated above, we found that compared with RPD, LPD demonstrated more specific gait impairments and sleep-related dysfunction. This study exhibited an effect of motor symptom laterality on the non-motor symptoms and gait performance in PD. This small but significant effect should be considered when evaluating and treating PD patients. However, more research, especially on the *de novo* group and longitudinal cohort, is needed to confirm these results.

## Data Availability Statement

The original contributions presented in the study are included in the article/supplementary material, further inquiries can be directed to the corresponding author.

## Ethics Statement

The studies involving human participants were reviewed and approved by Ethics committee of Nanjing Brain Hospital Affiliated to Nanjing Medical University. The patients/participants provided their written informed consent to participate in this study.

## Author Contributions

LZ, SZ, and MZ conceived and designed the research. LZ and JY obtained the fundings. YB, ZW, RG, XJ, BS, and JZ collected the data. YP, PX, JD, and JY conducted the data analysis. SZ and MZ drafted the manuscript. All authors contributed to the article and approved the submitted version.

## Conflict of Interest

The authors declare that the research was conducted in the absence of any commercial or financial relationships that could be construed as a potential conflict of interest.
